# A glutamate synthase mutant of *Bradyrhizobium* sp. strain ORS285 is unable to induce nodules on Nod factor-independent *Aeschynomene* species

**DOI:** 10.1038/s41598-021-00480-7

**Published:** 2021-10-22

**Authors:** Nico Nouwen, Clémence Chaintreuil, Joel Fardoux, Eric Giraud

**Affiliations:** Laboratoire des Symbioses Tropicales et Méditerranéennes (LSTM), UMR IRD/SupAgro/INRAE/Université de Montpellier/CIRAD - Campus de Baillarguet, Montpellier, France

**Keywords:** Microbiology, Plant sciences

## Abstract

The *Bradyrhizobium* sp. strain ORS285 is able to establish a nitrogen-fixing symbiosis with both Nod factor (NF) dependent and NF-independent *Aeschynomene* species. Here, we have studied the growth characteristics and symbiotic interaction of a glutamate synthase (GOGAT; *gltD::Tn5*) mutant of *Bradyrhizobium* ORS285. We show that the ORS285 *gltD::Tn5* mutant is unable to use ammonium, nitrate and many amino acids as nitrogen source for growth and is unable to fix nitrogen under free-living conditions. Moreover, on several nitrogen sources, the growth rate of the *gltB::Tn5* mutant was faster and/or the production of the carotenoid spirilloxanthin was much higher as compared to the wild-type strain. The absence of GOGAT activity has a drastic impact on the symbiotic interaction with NF-independent *Aeschynomene* species. With these species, inoculation with the ORS285 *gltD::Tn5* mutant does not result in the formation of nodules. In contrast, the ORS285 *gltD::Tn5* mutant is capable to induce nodules on NF-dependent *Aeschynomene* species, but these nodules were ineffective for nitrogen fixation. Interestingly, in NF-dependent and NF-independent *Aeschynomene* species inoculation with the ORS285 *gltD::Tn5* mutant results in browning of the plant tissue at the site of the infection suggesting that the mutant bacteria induce plant defence responses.

## Introduction

Rhizobia are able to establish a symbiotic interaction with leguminous plants. In this interaction, the rhizobium induces the plant to form a new organ, the nodule, infected by the bacteria which differentiate into dinitrogen fixing bacteroids. This symbiotic nitrogen fixation not only allows leguminous plant to grow on nitrogen poor soils but also has a high impact in natural environments as well as in agriculture as it is one of largest contributors of nitrogen input into the soil.

The *Bradyrhizobium* sp. strain ORS285 is a photosynthetic bacterium that forms a symbiotic interaction with tropical aquatic legumes of the *Aeschynomene* genus^1^. It infects plants at the base of lateral roots or the adventitious root primordia on the stem via colonization of intercellular spaces between the epidermis and outer cortex plant cells. After this initial intercellular infection, the bacteria penetrate some plant cells and these infected cortical cells start to divide rapidly and repeatedly to form the nodule primordium^2^. The ORS285–*Aeschynomene* interaction is a fascinating symbiotic model because, the ORS285 strain, differently to the other photosynthetic bradyrhizobia, such as ORS278 and BTAi1, has *nod* genes and is able to nodulate a broader host range of *Aeschynomene* species that differ by the requirement or not of Nod Factors (NFs) to trigger nodulation^3^.

**Figure 1:**
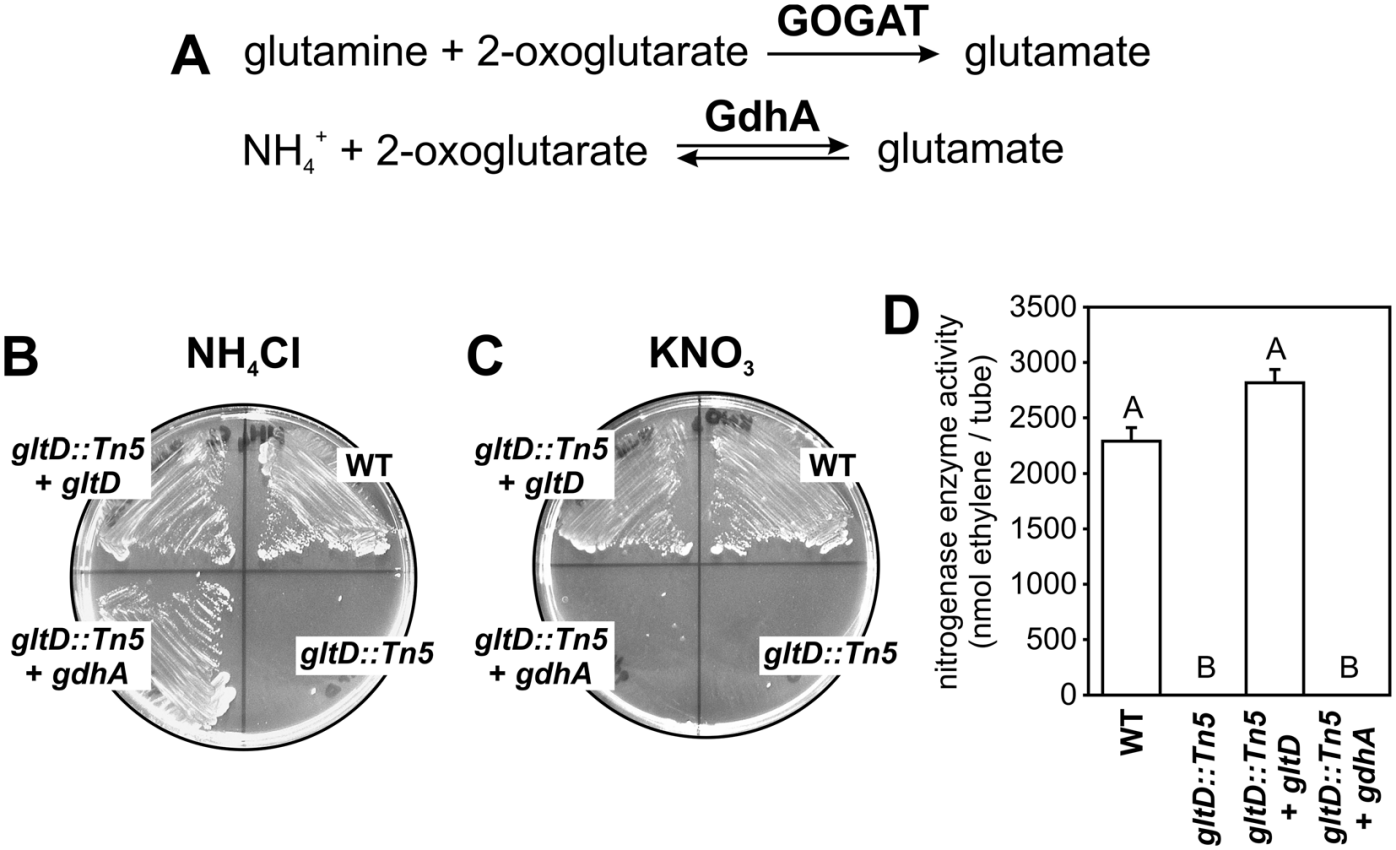
Growth and nitrogenase enzyme activity in free-living conditions of the *Bradyrhizobium* ORS285 *gltD::Tn5* mutant. (**A**) Biosynthesis of glutamate catalysed by the glutamate synthase (GOGAT) and glutamate dehydrogenase (GdhA) enzyme. Growth of *Bradyrhizobium* ORS285 and derivatives on Minimal medium agar plates containing (**B**) 10 mM NH_4_Cl and (**C**) 10 mM KNO_3_ as nitrogen source. (**D**) ORS285 *gltD::Tn5* mutant has no nitrogenase enzyme activity under free-living conditions. Ethylene production of ORS285 and derivatives grown for 5 days in vacuette tubes containing BNM-B agar (0.8% w/v) medium without nitrogen source and 10% acetylene gas. The mean amount of produced ethylene per tube (n = 4) is indicated. Error bars represent standard errors of the mean and letters represent conditions with significant difference according to the Tukey’s test (P < 0.05).

In a large screen for mutations that affect the symbiosis between the photosynthetic *Bradyrhizobium* strain ORS278 and the NF-independent *Aeschynomene* species, *Aeschynomene indica*, strains with Tn5 insertions into the *gltB* or *gltD* gene had a Nodule development defective (Ndv^-^) phenotype^4^. The *gltB* and *gltD* genes encode for the large and small subunit of the glutamate synthase, also known as glutamine 2-oxoglutarate amidotransferase (GOGAT) complex, respectively (Fig. 1A) Together with glutamine synthase (GS), the GS-GOGAT pathway plays an important role in the assimilation of NH_4_^+^ and de novo synthesis of glutamate in bacteria. Interestingly, whereas on plants inoculated with other ORS278 Ndv^-^ mutants, small onsets of nodule organogenesis (i.e. bumps at the base of lateral roots) were detected, these observations were very rare or completely absent on plants inoculated with the ORS278 *gltB::Tn5* and *gltD::Tn5* mutant strains. These results are quite surprising as in other rhizobium–legume interactions that depend on NFs, *gltB* and *gltD* mutants have no symbiotic defect^5,6^ or induce nodules that do not fix nitrogen (fix^-^ phenotype)^7–10^. To investigate this divergence, we have characterized the growth characteristics and symbiotic phenotype of a *Bradyrhizobium* ORS285 *gltD::Tn5* mutant strain with both NF-independent and NF-dependent *Aeschynomene* species. The results show that the absence of GOGAT activity has a drastic effect on the growth on different nitrogen sources, and that in contrast to the interaction with NF-independent *Aeschynomene* species, the ORS285 *gltD::Tn5* mutant strain is capable to induce nodules on NF-dependent host plants.

## Results

### Isolation and characterisation of a *Bradyrhizobium* ORS285 *gltD::Tn5* mutant and construction of strains restoring ammonium assimilation

A *Bradyrhizobium* ORS285 *gltD::Tn5* mutant was obtained by screening a random Tn5 insertion library of strain ORS285 for mutations that affect the symbiotic interaction with *Aeschynomene indica* and/or *Aeschynomene afraspera*, respectively. In one mutant strain with a symbiotic phenotype, the Tn5 insertion was found inside the *gltD* gene (BRAD285_V2_6186; Fig. 3A). The ORS285 *gltD::Tn5* mutant strain was unable to grow on minimal medium plates containing ammonium (NH_4_^+^) as sole nitrogen source. The growth of this strain in medium containing NH_4_^+^ could be restored when the WT *gltD* gene was re-introduced (strain ORS285 *gltD::Tn5* + *gltD*) or by introducing the *gdhA* gene of *E. coli* MG1655 (strain ORS285 *gltD::Tn5* + *gdhA)* (Fig. 1B). GdhA, encodes for a glutamate dehydrogenase, that is able to combine NH_4_^+^ and 2-oxoglutarate to form 1 molecule of glutamate (Fig. 1A). These results indicate that: (1) the Tn5 insertion into the *gltD* gene is responsible for the inability to use NH_4_^+^ as nitrogen source and (2) that the gene annotated as *gdhA* in ORS285 (BRAD285_V2_4857) is not transcribed or the gene product is not able to assimilate NH_4_^+^.

**Figure 2:**
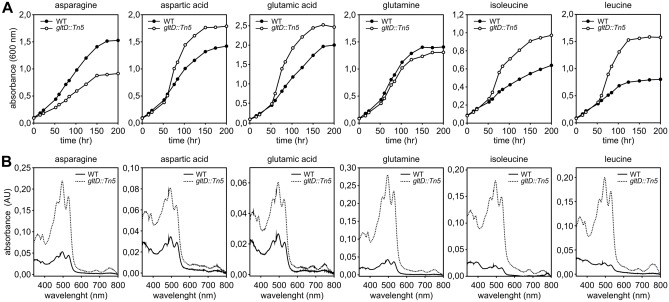
Growth and carotenoid production of the *Bradyrhizobium* ORS285 *gltD::Tn5* strain using different amino acids as nitrogen source. (**A**) Representative growth curves of *Bradyrhizobium* ORS285 and *Bradyrhizobium* ORS285 *gltD::Tn5* in minimal BNM-B medium containing different amino acids as nitrogen source. (**B**) Absorbance spectra of carotenoids extracted from 45 mg (wet weight) *Bradyrhizobium* ORS285 and *Bradyrhizobium* ORS285 *gltD::Tn5* cells at the end of the growth experiment shown in (**A**).

To analyse the effect of *gltD* inactivation on the usage of other nitrogen sources than NH_4_^+^, we have grown the ORS285 *gltD::Tn5* mutant on succinate minimal BNM-B medium plates containing different amino acids and other compounds as N-source and compared these to the growth of the WT strain, the ORS285 *gltD::Tn5* + *gltD* strain and the ORS285 *gltD::Tn5* + *gdhA* strain. Among the many compounds tested, the ORS285 *gltD::Tn5* mutant was only able to grow on plates containing the amino acids asparagine, aspartic acid, glutamic acid, glutamine, leucine and isoleucine as N-source (Table S1). To examine the utilization of these amino acids as N-source in more detail, we have also investigated the growth in liquid minimal medium. This showed that in medium containing aspartic acid, glutamic acid, isoleucine, and leucine as N-source, the ORS285 *gltD::Tn5* mutant strain grows faster and reaches a higher final optical density as compared to the WT strain (Fig. 2A). In addition, analysing the carotenoid content of bacterial cells showed that the ORS285 *gltD::Tn5* mutant strain on all tested amino acids, and in particular when using asparagine, glutamine, isoleucine and leucine as N-source, produced a drastically increased amount of spirilloxanthin as compared to the WT strain suggesting that the photosynthetic activity is boosted in the mutant. (Fig. 2B). These experiments show that besides the inability to use certain N-sources, the nitrogen and most probably carbon metabolism of the ORS285 *gltD::Tn5* mutant strain is drastically different from the WT strain. In the experiments, also a remarkable observation was made with the mutant strain expressing *E. coli* GdhA. The presence of the *E. coli gdhA* gene enabled the *gltD::Tn5* mutant strain to use almost the same N-sources as WT ORS285 (Table S1). The only exceptions were the growth on KNO_3_ (Fig. 1C; Table S1) and reduction of atmospheric dinitrogen (N_2_) as measured by the acetylene reduction assay (ARA) (Fig. 1D). This suggests that with these two N-sources, the concentration of intracellular NH_4_^+^ (and/or 2-oxoglutarate) is not sufficient to form glutamate in the reaction catalysed by *E. coli* GdhA.

### Expression of *gltB/D* operon in the presence of different N-sources and in symbiosis

**Figure 3:**
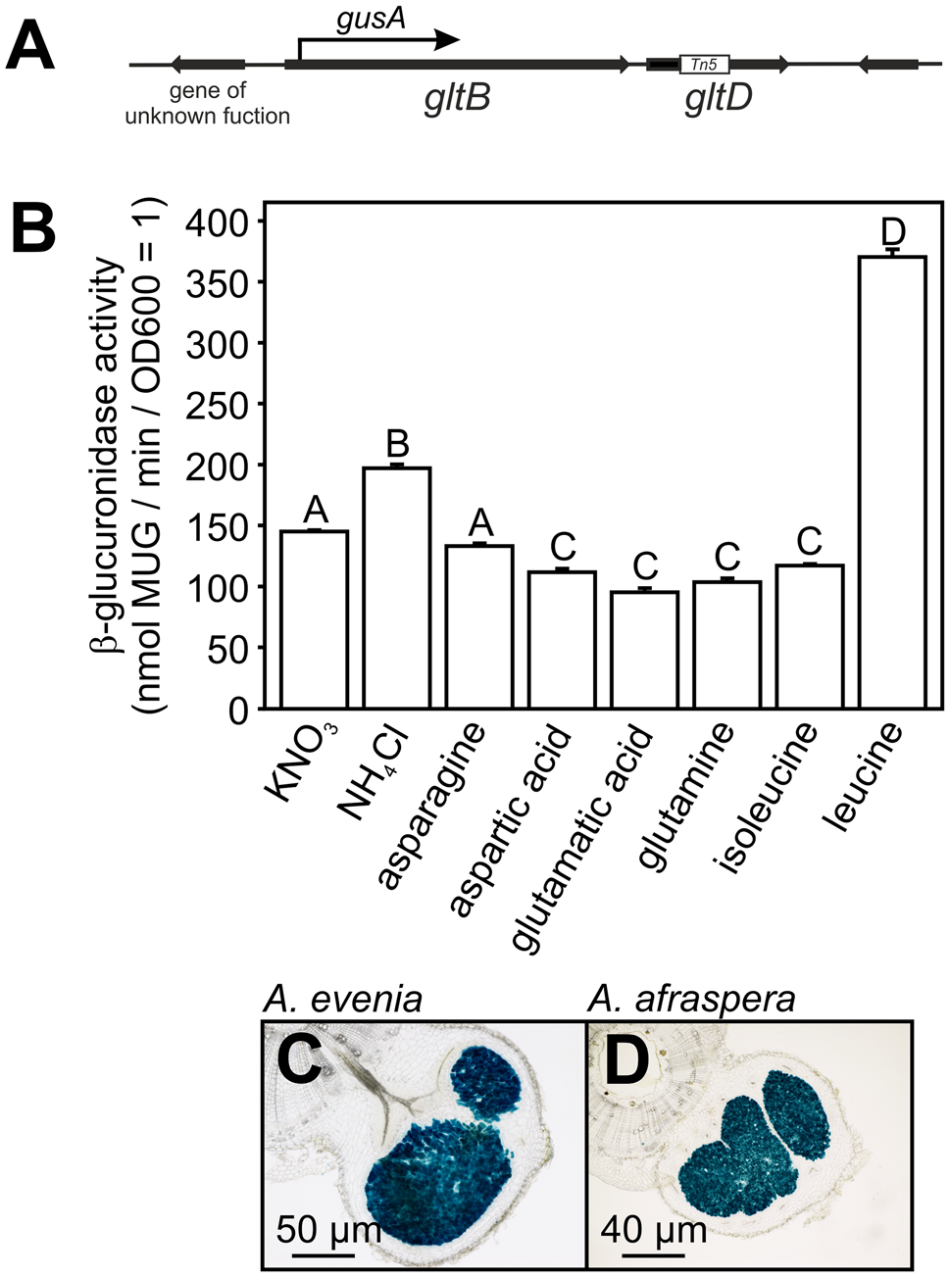
Activity of the *Bradyrhizobium* ORS285 GOGAT promoter under free-living and symbiotic conditions. (**A**) Schematic representation of the genomic region containing the *gltB*-*gltD* operon in *Bradyrhizobium* ORS285. The insertion of the Tn5 transposon in the *gltD* gene is indicated by a white block and the point of the transcriptional fusion with the *gusA* gene in the P_GOGAT_ reporter strain with and arrow. (**B**) β-glucuronidase activity of *Bradyrhizobium* ORS285 P_GOGAT_-gusA cells grown for 48 h in minimal BNM-B medium using the indicated nitrogen source. The results are from one representative experiment with three technical replicates for each experimental condition. Error bars represent standard errors of the mean and letters represent conditions with significant difference according to the Tukey’s test (P < 0.05). GOGAT promoter activity in nodules of (**C**) NF-independent *A. evenia* and (**D**) NF-dependent *A. afraspera* plants. *A.* *evenia* and *A. afraspera* plants were infected with the *Bradyrhizobium* ORS285 P_GOGAT_-*gusA* reporter strain and at 15 dpi, the β-glucuronidase activity in 70 µM nodule sections was determined by X-Gluc staining.

To measure *gltB/D* expression, we have constructed a reporter strain in which *gusA* gene was placed under the control of the promoter region of the *gltB/D* operon (Fig. 3A). This reporter strain (ORS285 P_GOGAT_-*gusA*) was subsequently grown in the presence of different nitrogen salts (NH_4_^+^/NO_3_^-^) or amino acids that allow growth of the ORS285 *gltD::Tn5* mutant strain and the β-glucuronidase activity was measured after 48 h of incubation at 28 °C. Between the majority of analysed N-sources there is only a slight (but statistical relevant) difference in β-glucuronidase activity (Fig. 3B). An exception are cells grown in the presence of the amino acid leucine for which the measured β-glucuronidase activity was 2 to 3.5 -fold higher as compared to cells grown in the presence of other N-sources (Fig. 3B).

To investigate the expression of the *gltB/D* operon during symbiosis, the ORS285 P_GOGAT_-*gusA* strain was used to infect the NF-independent and NF-dependent *Aeschynomene* species *A. evenia* and *A. afraspera*, respectively. Fifteen days post infection, some nodules were sliced and nodule sections were incubated with the β-glucuronidase substrate 5-bromo-4-chloro-3-indolyl-beta-d-glucuronic acid (X-Gluc). Blue staining of the central nodule tissue showed that the *gltB/D* operon is expressed in mature nodules of both *A. evenia* and *A. afraspera* plants (Fig. 3C, D). Although X-gluc staining is a qualitative measurement of gene expression, it should be noted that no significant difference in the colour could be detected by eye between the two species.

### Glutamate synthase (GOGAT) activity is required for nodule organogenesis in NF-independent *Aeschynomene evenia* plants

**Figure 4:**
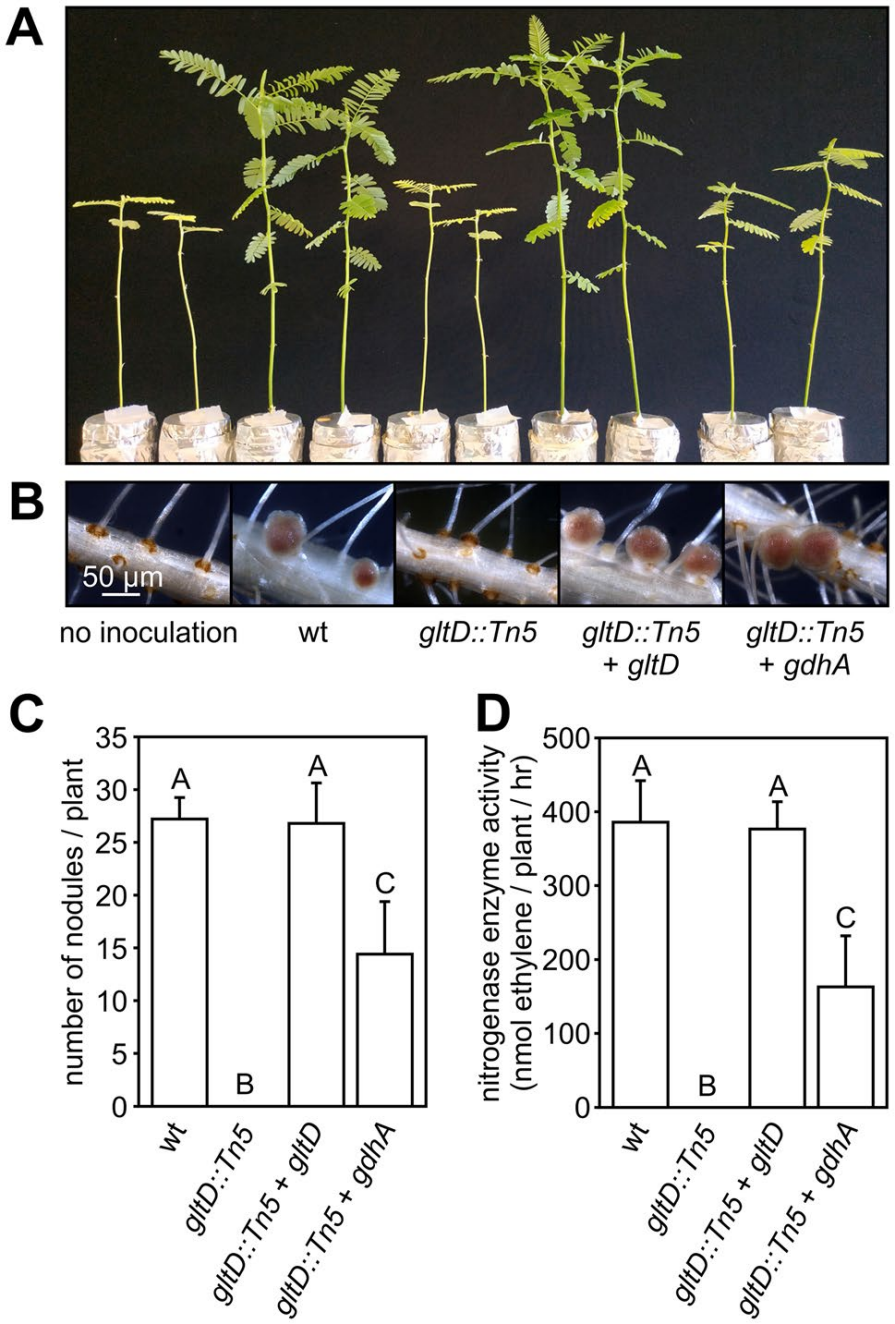
The *Bradyrhizobium* ORS285 *gltD::Tn5* mutant does not induce nodules on *Aeschynomene evenia* (CIAT22838) plants. (**A**) Comparison of the growth of *A. evenia* plants inoculated with ORS285, ORS285 *gltD::Tn5*, ORS285 *gltD::Tn5* + *gltD*, and ORS285 *gltD::Tn5* + *gdhA*. Non-inoculated plants (ni) were used as control. (**B**) Roots of *A. evenia* plants inoculated with ORS285, ORS285 *gltD::Tn5*, ORS285 *gltD::Tn5* + *gltD*, and ORS285 *gltD::Tn5* + *gdhA*. Note the absence of nodules on *A. evenia* plants inoculated with the ORS285 *gltD::Tn5* strain. (**C**) Number of root nodules on *A. evenia* plants inoculated with ORS285, ORS285 *gltD::Tn5*, ORS285 *gltD::Tn5* + *gltD*, and ORS285 *gltD::Tn5* + *gdhA*, respectively. The mean number of nodules per plant (n = 5) at 21 dpi is presented. (**D**) Acetylene reducing activity of *A. evenia* plants inoculated with ORS285, ORS285 *gltD::Tn5*, ORS285 *gltD::Tn5* + *gltD*, and ORS285 *gltD::Tn5* + *gdhA* at 21 dpi. The mean amount of produced ethylene per hour and per plant (n = 5) is indicated. In (**C**) and (**D**) error bars represent standard errors of the mean and letters represent conditions with significant difference according to the Tukey’s test (P < 0.05).

*Bradyrhizobium* ORS285 has a relative broad host range and dependent on the plant species uses a NF-independent or NF-dependent mechanism to establish a symbiotic interaction with *Aeschynomene* legumes^3^. To investigate the effect of the GOGAT mutation in the symbiotic interaction with a NF-independent host plant, we infected our model species *Aeschynomene evenia* with the WT, and ORS285 *gltD::Tn5*, ORS285 *gltD::Tn5* + *gltD* and ORS285 *gltD::Tn5* + *gdhA* mutants. As previously observed for a GOGAT mutant in ORS278 on *A. indica*, the ORS285 *gltD::Tn5* mutant failed to induce nodules on *A. evenia* . The most unexpected observation is the fact that the complementation of this mutant with *gltD* or *gdhA* resulted in two completely distinct phenotypes. Whereas introducing the *gltD* gene into the ORS285 *gltD::Tn5* mutant completely restored the plant growth, the plants inoculated with the ORS285 *gltD::Tn5* + *gdhA* mutant showed reduced growth and a mild form of foliar chlorosis (Fig. 4A). Analysis of the roots showed that plants inoculated with the ORS285 *gltD::Tn5* mutant did not contain nodules, whereas the roots of plants inoculated with the ORS285 *gltD::Tn5* + *gdhA* mutant contained WT-like nodules (pink/rose colour) but the number was approximately half as compared to plants inoculated with the WT strain (Fig. 4B, C). In line with the visual observations, the acetylene reduction assay (ARA) showed that plants infected with the ORS285 *gltD::Tn5* mutant had no nitrogenase enzyme activity, and those infected with ORS285 *gltD::Tn5* + *gdhA* mutant an activity that was approximately half as observed with plants infected with the WT strain (Fig. 4D). Observations of the roots during the experiments showed that on plants inoculated with the ORS285 *gltD::Tn5* + *gdhA* mutant the first full-sized nodules appeared at 11–14 dpi whereas on plants inoculated with the WT strain this was at 3–5 dpi. As the ORS285 *gltD::Tn5* + *gdhA* mutant had no nitrogenase enzyme activity under free-living conditions (Fig. 1D), we analysed if the formed nodules on ORS285 *gltD::Tn5* + *gdhA* inoculated plants were not due to a reversion of the *gltD::Tn5* mutation. Bacteria isolated from nodules at 23 dpi were kanamycin resistant and able to grow on minimal medium plates containing NH_4_Cl as N-source (Fig. S1A, D). However in contrast to the original ORS285 *gltD::Tn5* + *gdhA* mutant strain, bacteria re-isolated from the nodules were able to grow on plates containing KNO_3_ as nitrogen source (Fig. S1C). These results indicate that the observed nodules and nitrogenase enzyme activity on plants inoculated with the *gltD::Tn5* + *gdhA* mutant are likely the result of a reversion of the *gltD* mutation. Plants inoculated with the mutant in which the WT *gltD* gene was re-introduced (ORS285 *gltD::Tn5* + *gltD* ) were in all analysed characteristics indistinguishable from plants inoculated with the WT strain (Fig. 4A-D).

**Figure 5:**
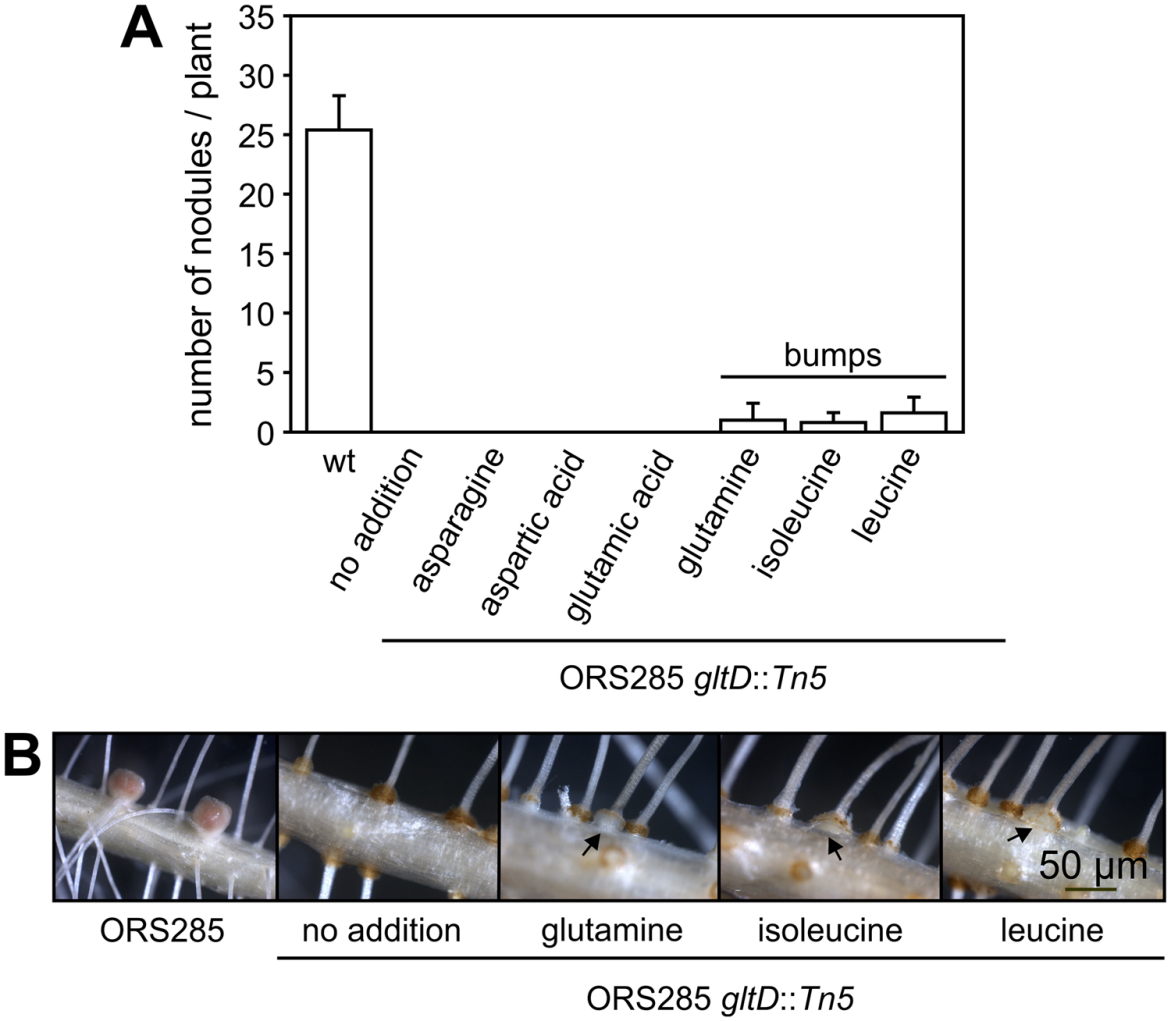
Amino acid supplementation does not restore the symbiotic phenotype of the *Bradyrhizobium gltD::Tn5* mutant with *A. evenia* plants. (**A**) *A. evenia* plants were inoculated with the *Bradyrhizobium* ORS285 *gltD::Tn5* mutant in the presence of 0.5 mM of the indicated amino acid and at 21 dpi the number of nodules (cq. bumps) was determined. (**B**) mature nodules induced by wild-type ORS285 and small bumps induced by the ORS285 *gltD::Tn5* mutant strain in the presence of 0.5 mM glutamine, isoleucine or leucine on *A. evenia* roots.

Growth experiments with the ORS285 *gltD::Tn5* mutant showed that it can use only a few amino acids as nitrogen source (Table S1; Fig. 2A). To investigate if these amino acids can complement the symbiotic deficiency of the ORS285 *gltD::Tn5* mutant, *A. evenia* plants were inoculated with the ORS285 *gltD::Tn5* mutant strain in the presence of 0.5 mM of these different amino acids added to the plant growth medium. The addition of all these amino acids did not complement the symbiotic phenotype of the ORS285 *gltD::Tn5* mutant. Nevertheless, in the case of glutamine, isoleucine or leucine addition, some of the *A. evenia* plants contained bump-like structures at the base of lateral roots whereas such structures were not observed when asparagine, aspartic acid or glutamic acid were added (Fig. 5A, B).

**Figure 6:**
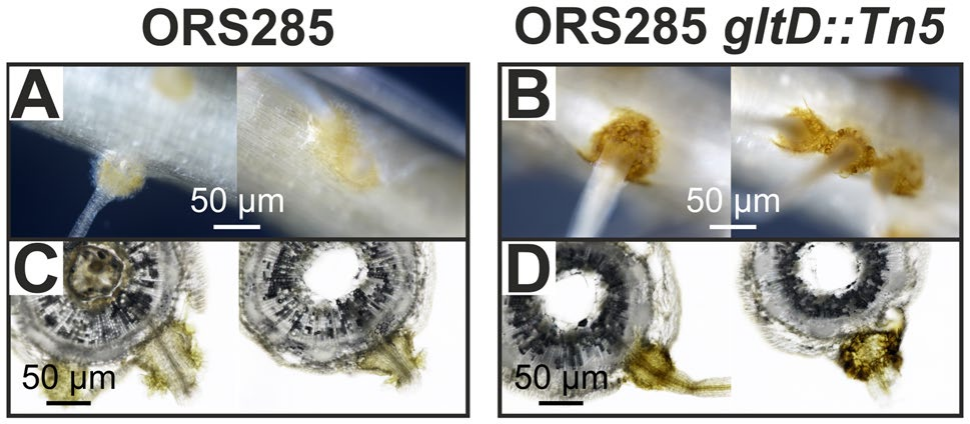
More intense browning of auxiliary root hairs on *A. evenia* plants inoculated with the *Bradyrhizobium gltD::Tn5* mutant. Auxiliary root hairs of *A. evenia* plants inoculated with the (**A**) wild-type and (**B**) *Bradyrhizobium gltD::Tn5* mutant strain at 5 dpi. 200 µM coupes of root segments at 5 dpi to visualize the auxiliary root hairs of *A. evenia* plants inoculated with the (**C**) wild-type and (**D**) *Bradyrhizobium gltD::Tn5* mutant strain.

When examining the roots of ORS285 *gltD::Tn5* infected plants, we noticed that the auxiliary root hairs at the base of a lot of lateral roots, which correspond to the site of infection, had a more intense brown colour as compared to the ones present on plants inoculated with the WT strain (Fig. 6A, C vs B, D). This is indicative for the accumulation of polyphenol compounds generally associated with plant defence reactions, as previously described in other legumes^11^.

NF-independent *Aeschynomene* species form a monophyletic clade that can be divided into four sub-groups^12^. To investigate whether the symbiotic requirement for GOGAT activity as found for *A. evenia* is a generality within this clade, we infected one species of the three other sub-groups (*A. sensitiva*, *A. deamii*, *A. tambacoudensis*) with the *gltD::Tn5* mutant strain. With all analysed species, plants infected with the *gltD::Tn5* mutant strain displayed: (1) nitrogen starvation symptoms (Fig. S2–S4, A); (2) the absence of nodules on their roots (Fig. S2–S4 B, C); (3) no nitrogenase enzyme activity (Fig. S2–S4, D). These data indicate that an active glutamate synthase complex (GOGAT) is obligatory for the induction of nodules on roots of NF-independent *Aeschynomene* species.

### In NF-dependent *Aeschynome* species the absence of glutamate synthase (GOGAT) activity results in a Fix^-^ phenotype

**Figure 7:**
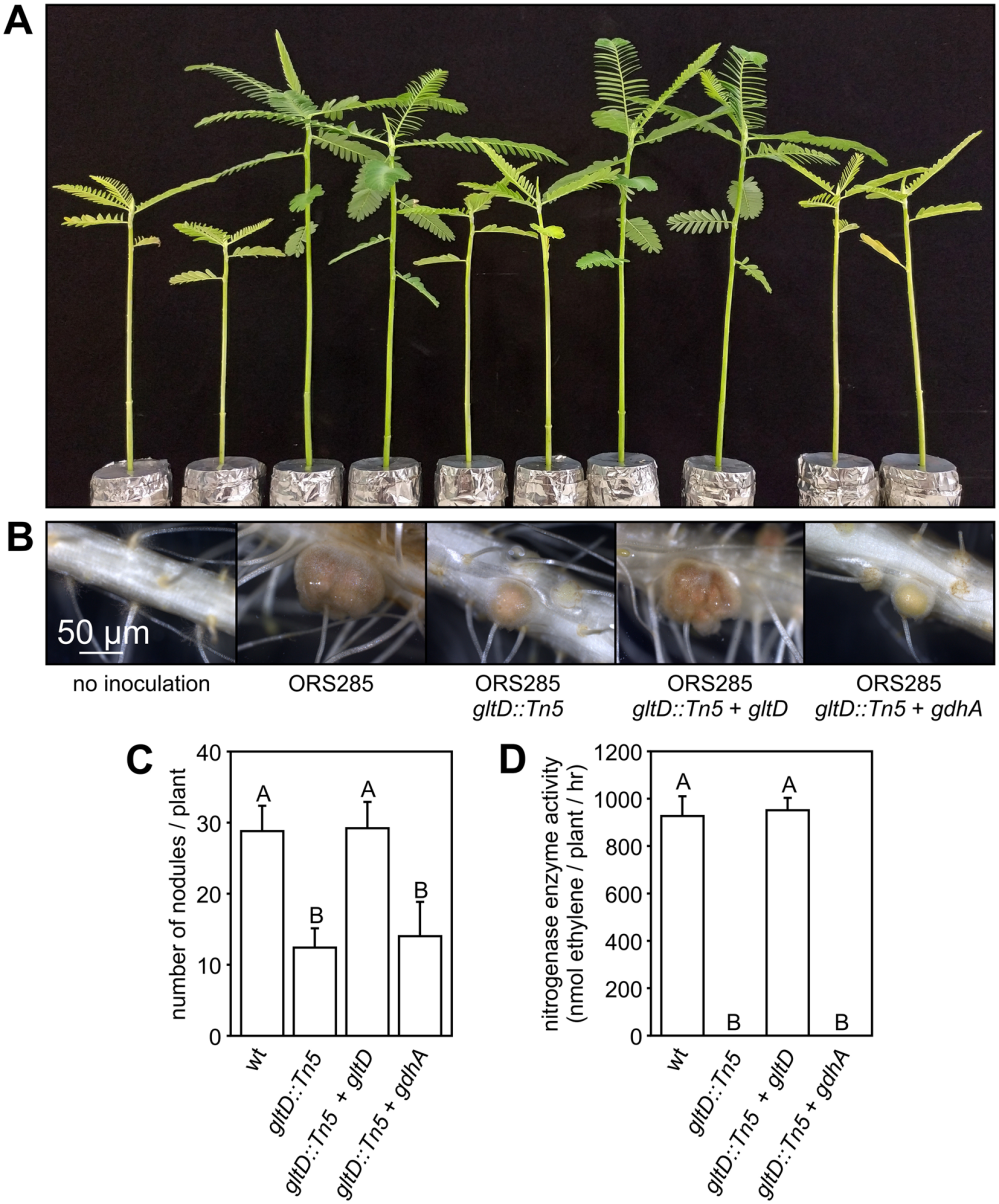
The *Bradyrhizobium* ORS285 *gltD::Tn5* mutant does induce nodules on *Aeschynomene afraspera* (LSTM #1) plants. (**A**) Comparison of the growth of *A. afraspera* plants inoculated with ORS285, ORS285 *gltD::Tn5*, ORS285 *gltD::Tn5* + *gltD*, and ORS285 *gltD::Tn5* + *gdhA*. Non-inoculated plants (ni) were used as control. (**B**) Roots of *A. afraspera* plants inoculated with ORS285, ORS285 *gltD::Tn5*, ORS285 *gltD::Tn5* + *gltD*, and ORS285 *gltD::Tn5* + *gdhA*. (**C**) Number of root nodules on *A. afraspera* plants inoculated with ORS285, ORS285 *gltD::Tn5*, ORS285 *gltD::Tn5* + *gltD*, and ORS285 *gltD::Tn5* + *gdhA*, respectively. The mean number of nodules per plant (n = 5) at 21 dpi is presented. (**D**) Acetylene reducing activity of *A. afraspera* plants inoculated with ORS285, ORS285 *gltD::Tn5*, ORS285 *gltD::Tn5* + *gltD*, and ORS285 *gltD::Tn5* + *gdhA* at 21 dpi. The mean amount of produced ethylene per hour and per plant (n = 5) is indicated. In (**C**) and (**D**) error bars represent standard errors of the mean and letters represent conditions with significant difference according to the Tukey’s test (P < 0.05).

We also investigated the role of GOGAT activity in the symbiotic interaction with a NF-dependent host plant. For this, we infected the model species *A. afraspera* with the different mutant strains and observed the effect on plant growth, nodule number and nitrogenase enzyme activity at 20 dpi. Non-inoculated, ORS285 *gltD::Tn5* and ORS285 *gltD::Tn5* + *gdhA* infected plants had clear nitrogen starvation signs such as reduced plant growth and foliar chlorosis (Fig. 7A). In contrast to what observed with NF-independent *Aeschynomene* species, the ORS285 *gltD::Tn5* mutant strain induced nodules on the roots of *A. afraspera* plants (Fig. 7B). However, the number of nodules on ORS285 *gltD::Tn5* inoculated plants was about half the number as compared to WT inoculated plants and their colour was light-pink/white in contrast to red/pink as found for WT nodules (Fig. 7B, C). In line with these observations, no nitrogenase enzyme activity was detected in the ARA assay on plants inoculated with the ORS285 *gltD::Tn5* mutant (Fig. 7D). Exactly the same observations were made with plants that had been inoculated with the ORS285 *gltD::Tn5* + *gdhA* mutant, whereas the ORS285 *gltD::Tn5* + *gltD* mutant behaved like the WT strain.

**Figure 8:**
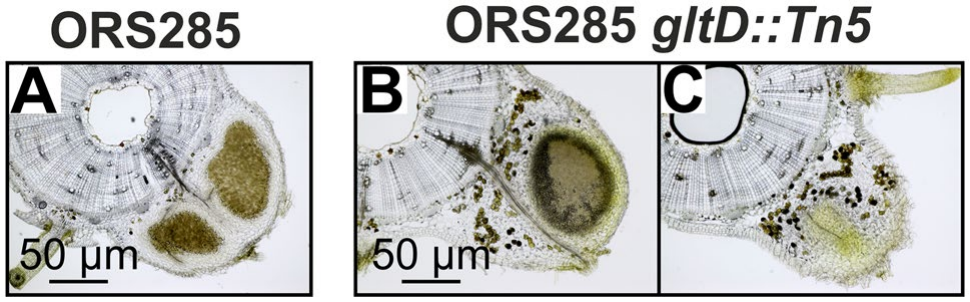
*A. afraspera* nodules induced by the *Bradyrhizobium* ORS285 *gltD::Tn5* mutant contain many plant cells with a gold-brown substance. 70 µM nodule sections of *A. afraspera* plants inoculated with (**A**) wild-type ORS285 and (**B**), (**C**) ORS285 *gltD::Tn5* mutant. Note the abundant presence of plant cells containing a gold-brown substance in nodule sections of plants inoculated with the ORS285 *gltD::Tn5* mutant strain.

Cytological analysis of nodules induced by ORS285 *gltD::Tn5* strain, showed that the bacterial infection of nodules induced by the ORS285 *gltD::Tn5* mutant strain was relatively heterogeneous (Fig. 8B, C). Some nodules contained a large infected central tissue like as found in WT nodules (Fig. 8, A *vs* B), whereas in other nodules this “zone” was absent or small (Fig. 8C). Moreover, in sections of the ORS285 *gltD::Tn5* nodules, and in particular in the ones without a central infected zone, numerous plant cells having a typical brown colour were observed. This suggests that the infection by the ORS285 *gltD::Tn5* mutant strain induced also defence responses in *A. afraspera* plants but probably to a less extent than observed in *A. evenia*.

We also tested the GOGAT mutant with another NF-dependent *Aeschynomene* species, *A. nilotica*. With this plant species, the *gltD::Tn5* mutant had exactly the same symbiotic phenotype, i.e. nitrogen starvation symptoms, reduced nodule number and no nitrogenase enzyme activity, as observed with *A. afraspera* plants (Fig. S5, A–D). In addition, a light brown colour at the outer surface of the nodules and presence of plant cells with an intense brown colour inside the nodule tissue were observed on plants infected with the ORS285 *gltD::Tn5* strain (Fig. S5, E *vs*. F, G). Thus upon interaction with members of the group of NF-dependent *Aeschynomene* species, the ORS285 *gltD::Tn5* mutant is able to induce nodule organogenesis and to infect the plant tissue but this interaction does not lead to a nitrogen fixing symbiosis.

## Discussion

In a previous Tn5 mutagenesis study to identify genes important for the NF-independent symbiotic interaction of photosynthetic *Bradyrhizobium* ORS278 with *Aeschynomene indica*, we observed that strains with Tn5 insertion into the *gltB* or *gltD* gene had the strongest ndv^-^ (nodule development) phenotype^4^. In this report, we have characterized a glutamate synthase (GOGAT; *gltD::Tn5*) mutant of *Bradyrhizobium* ORS285 and its symbiotic properties with NF-dependent and NF-independent *Aeschynomene* species. As shown for GOGAT mutants of other rhizobia^5–7,9,10^, the ORS285 *gltD::Tn5* mutant is unable to use ammonium, nitrate and several amino acids as nitrogen source (Table S1). In bacteria, ammonium assimilation can take place via the glutamine synthase (GS) –glutamate synthase (GOGAT) pathway or via the enzyme glutamate dehydrogenase (GdhA) (Fig. 1A). Genome analysis showed that the ORS285 strain, in contrast to many other rhizobia, has a glutamate dehydrogenase (GDH) homologue (BRAD285_V2_4857) which displays 48% of identity with the GDH of the hyperthermophilic bacterium *Thermotoga maritima*. However, despite the presence of this *gdh* gene and transcription under free-living conditions, the *gltD::Tn5* mutant strain is incapable to use NH_4_^+^ as nitrogen source. Interestingly, when the *E. coli gdhA* gene was introduced into the ORS285 *gltD::Tn5* mutant, the strain was capable to grow on NH_4_^+^ and amino acids as found for the wild-type strain (Fig. 1B; Table S1). This indicates that the NH_4_^+^ and 2-oxoglutarate concentrations in the ORS285 cell are sufficient high for ammonium assimilation via the GDH pathway but that the BRAD285_V2_4857 gene product is unable to do so and probably only has glutamate dehydrogenase activity. Surprisingly, the *E. coli gdhA* gene was unable to restore growth of the *gltD::Tn5* mutant on NO_3_^-^ plates (Fig. 1C) and no nitrogenase enzyme activity was detected in the acetylene reduction (ARA) assay under free-living conditions (Fig. 1D). Enzymes involved in nitrate and atmospheric nitrogen reduction are induced and have a complex regulation mechanism that also involve glutamine and 2-oxoglutarate concentrations in the cell^13,14^. We hypothesize that in the absence of GOGAT activity, the metabolism and glutamine/2-oxoglutarate concentrations in the cell are drastically altered and that this causes that the ORS285 *gltD::Tn5* mutant is incapable to induce the *nif* and nitrate reductase genes despite the presence of *E. coli gdhA*. In line with an altered metabolism in the ORS285 *gltD::Tn5* mutant, is the observation that this strain grows faster and produces drastically more photosynthetic pigment as compared to the wild-type ORS285 strain when using certain amino acids as nitrogen source (Fig. 2A, B). Thus besides the inability of the *gltD::Tn5* mutant to assimilate ammonium and to synthesize glutamate via the GS-GOGAT pathway, the regulation of nitrogen and carbon metabolism in the mutant bacteria seems to be largely altered.

In this study, we show that GOGAT activity in ORS285 is absolutely required for nodule organogenesis in the interaction with NF-independent *Aeschynomene* species. In other rhizobium-legume interactions, different symbiotic phenotypes of GOGAT mutants have been described, but none has been that drastic as observed for the *gltD* and *gltB/D* mutants of ORS285 (this study) and ORS278^4^, respectively, upon interaction with NF-independent *Aeschynomene* legumes. Whereas GOGAT mutants of *Bradyrhizobium diazoefficiens* and *Azorhizobium caulinodans* are forming nodules that are unable to fix nitrogen (O'Gara et al., 1984; Hilgert et al., 1987), a *Sinorhizobium meliloti* mutant does not have a symbiotic phenotype. Moreover, the absence of GOGAT activity in *Sinorhizobium etli* results in a more efficient symbiosis (Castillo et al., 2000). As all above described symbiotic interactions depend on NFs, the nodule organogenesis minus phenotype of the ORS285 *gltD::Tn5* mutant with certain *Aeschynomene* species could thus be related to the NF-independent character of the symbiotic interaction. In line with this hypothesis are the observations that the ORS285 *gltD::Tn5* mutant is able to induce nodules on the NF-dependent *Aeschynomene* species *A. afraspera* and *A. nilotica* (Fig. 7; Fig. S5) However, the induced nodules on the NF-dependent *Aeschynomene* species are unable to fix atmospheric nitrogen (Fig. 7D; Fig. S5, D). The latter is not so surprising as the ORS285 *gltD::Tn5* mutant also did not have nitrogenase enzyme activity under free-living conditions (Fig. 1D).

Growth experiments showed that the ORS285 *gltD::Tn5* mutant can only use a limited number of amino acids as nitrogen source (Table S1). As these amino acids could be limiting in the plant environment, we did experiments were we added amino acids that can be used by the ORS285 *gltD::Tn5* mutant to the plant growth medium (0.5 mM final concentration) (Fig. 5). With some amino acids (glutamine, isoleucine, leucine) this resulted in the rare formation of very small bumps on some of the ORS285 *gltD::Tn5* inoculated plants. We therefore believe that a reduced growth of the ORS285 *gltD::Tn5* mutant is not the main cause of the nodule organogenesis minus phenotype as observed with NF-independent *Aeschynomene* species. Observing the auxiliary root hairs of *A. evenia* plants inoculated with the ORS285 *gltD::Tn5* mutant, we noticed at a few days post infection a more intense browning of the axillary root hairs as compared to plants inoculated with the wild-type ORS285 (Fig. 6). This suggests that plants inoculated with the ORS285 *gltD::Tn5* mutant respond with an accumulation of polyphenols at the site of colonization and infection by the bacterium. Interestingly, an intense browning of multiple plant cells inside nodules was also observed on *A. afraspera* and *A. nilotica* plants inoculated with the ORS285 *gltD::Tn5* mutant (Fig. 8B, C; Fig. S5, F, G). Moreover, whereas mutant rhizobia unable to fix atmospheric nitrogen normally induce more nodules on plants as compared to the wild-type strain, the ORS285 *gltD::Tn5* mutant strain induces about half the amount of nodules on NF-dependent *Aeschynomene* species as compared to wild type ORS285 (Fig. 7C; Fig. S5, C). We therefore hypothesize that ORS285 *gltD::Tn5* bacteria induce a plant immune response in *Aeschynomene* plants, which is sufficient to completely inhibit nodule organogenesis and infection in NF-independent *Aeschynomene* species, but which, likely due to the perception of NFs, is to some extent suppressed in NF-dependent *Aeschynomene* species.

At this stage it is difficult to point what in the ORS285 *gltD::Tn5* mutant causes the immune response in *Aeschynomene* plants. The growth experiments showed that the (nitrogen) metabolism in ORS285 *gltD::Tn5* mutant bacteria is drastically different than in wild-type bacteria. This may lead to different proteins and or molecules at the cell surface of ORS285 *gltD::Tn5* mutant bacteria that are incompatible with a symbiotic interaction. Alternatively, a more restricted metabolism due to the absence of GOGAT enzyme activity in the ORS285 *gltD::Tn5* mutant could lead that the bacteria are not capable to adapt to the symbiotic conditions leading to bacterial cell lysis and subsequent induction of a plant immune response.

## Material and methods

### Bacterial strains and growth conditions

Tables of strains and plasmids used in this study can be found in the Supporting information (Tables S2, S3). *Bradyrhizobium* ORS285^15^ and derivatives were grown at 28 °C in modified yeast extract mannitol medium (YM^16^) or a minimal BNM-B medium^17^ with 10 mM succinate as carbon source and 10 mM ammonium chloride as nitrogen source, unless otherwise indicated. When required, ampicillin (50–100 µg/ml), kanamycin (50–120 µg/ml), or cefotaxime (20 µg/ml) were added to the growth medium.

### Isolation of a *Bradyrhizobium* ORS285 *gltD::Tn5* mutant

A *Bradyrhizobium* ORS285 Tn5 transposon library was constructed via a biparental mating protocol as described by^18^ using *E. coli* BW20767 containing plasmid pCRS487 harbouring the mini-transposon mTn5-GNm as donor strain^19^. Approximately five thousand kanamycine resistant clones were isolated from several independent matings and analysed for their symbiotic phenotype on *A. indica* and *A. afraspera* plants as described in Bonaldi et al.^4^. Mutants showing a symbiotic phenotype were retained and the Tn5 insertion site was determined. One of the isolated mutants had the mTn5-GNm transposon inserted into the *gltD* gene.

### Complementation of the ORS285 *gltD::Tn5* mutant

Complementation of growth of the ORS285 *gltD::Tn5* mutant on minimal medium plates containing ammonium chloride as nitrogen source was tested using two different approaches: 1) restoring the mutant *gltD* gene with a wild-type copy of the gene, 2) introduction of the glutamate dehydrogenase gene (*gdhA*) of *E. coli* MG1655. The *Bradyrhizobium* ORS285 *gltD* and *E. coli* MG1655 *gdhA* DNA regions were PCR amplified using primers that can be found in Table S4 of the supplementary information and cloned into pGEM-T Easy (Promega) and transformed into thermocompetent *E. coli* XL2-Blue cells (Agilent). Correct clones were verified by sequence analysis. The fragment containing the complete ORS285 *gltD* gene in pGEM-T Easy was excised with *Spe*I-*Bam*HI and ligated into suicide vector pJG194^20^ digested with *Spe*I-*Bam*HI. After transformation into *E. coli* XL2 Blue cells, correct clones were selected via kanamycin resistance (50 µg/ml) and subsequent DNA restriction enzyme analysis. The *E. coli* MG1655 *gdhA* gene in pGEM-T Easy was excised with *Bam*HI-*Eco*RI and ligated into suicide vector pJG194-4694-*miaA*^17^ digested with *Bam*HI-*Eco*RI. This places the *E. coli* MG1655 *gdhA* gene under control of a promoter that is constitutively expressed in *Bradyrhizobium* ORS285. pJG194 plasmids containing the ORS285 *gltD* and *E. coli gdhA* gene, respectively, were transformed into CaCl_2_ competent *E. coli* S17-1 cells^21^ and mobilized into the *Bradyrhizobium* ORS285 *gltD::Tn5* mutant using the biparental mating protocol as previously described^17^. After mating, ORS285 *gltD::Tn5* bacteria in which the plasmid has been integrated into the chromosome were selected on minimal medium plates containing 120 µg**/**ml kanamycine, 20 µg**/**ml cefotaxime and 10 mM ammonium chloride as nitrogen source. Correct insertion of the plasmids was verified by PCR.

### Construction of a GOGAT reporter strains of *Bradyrhizobium* ORS285

To construct a GOGAT reporter strain, the 576 bp region upstream of the *gltB-gltD* was amplified by PCR using the primers indicated in Table S3 (Supplementary information), cloned into pGEM-T Easy and transformed into *E. coli* XL2-Blue cells. After verification by sequence analysis, the promoter fragment was excised with *Sal*I-*Bam*HI and ligated into suicide vector pJG194-PnodA-*gusA*^22^ digested with the same enzymes. After transformation into *E. coli* XL2 Blue cells, correct clones were selected as described above. Plasmids were transformed into CaCl_2_ competent *E. coli* S17-1 cells^21^ and mobilized into *Bradyrhizobium* ORS285 using the biparental mating^17^. Correct insertion of the plasmids was verified by PCR.

### Expression of the *gltB-gltD* operon in the presence of different N-sources

The *Bradyrhizobium* ORS285 P_GOGAT_-*gusA* reporter strain was used to measure the expression of the *gltB-gltB* operon when bacteria are grown in minimal BNM-B medium^17^ containing potassium nitrate, ammonium chloride or different amino acids (10 mM final concentration) as nitrogen source. *Bradyrhizobium* ORS285 P_GOGAT_-*gusA* was grown YM medium for three days at 28 °C. The bacteria were harvested, washed two times with BNM-B medium without carbon and nitrogen source and subsequently diluted into BNM-B medium (OD600 = 0.1) containing 10 mM succinate as carbon source and the indicated nitrogen source (10 mM). After 48 h of growth at 28 °C, the absorbance at 600 nm of the cultures were determined and the β-glucuronidase activities were measured according to the method of Miller^23^ using 4-methylumbelliferyl-β-d-glucuronide (Sigma-Aldrich) as substrate. Experiments were at least repeated twice with three technical replicates in one experiment.

### In vitro nitrogenase enzyme activity

To measure the nitrogenase enzyme activity under free-living conditions, *Bradyrhizobium* ORS285 and derivatives were grown in 2 ml BNM-B 0.8% agar medium in 9 ml vacuette tubes (Greiner bio-one; ref: 455001). To circumvent overpressure, 0.9 ml of air was removed before injecting the same volume of 100% acetylene. The cultures were incubated at 28 °C and at 5 days 1 ml gas samples were analysed for ethylene production by gas chromatography^24^.

### Carotenoid extraction

After 8 days of growth at 28 °C in minimal BNM-B medium containing different amino acids as nitrogen source, bacteria were harvested by centrifugation (15 min 4000×*g*). After complete removal of the culture supernatant, the weight of the cell pellet was determined, whereafter carotenoids were extracted by dissolving 45 mg (wet weight) cells in a 1 ml acetone/methanol (7:2) mixture. After 5 h incubation with regular mixing on ice, the mixture was centrifuged for 10 min at 20,000×*g* in an Eppendorf centrifuge, whereafter the supernatant was collected. To determine the amount of carotenoids in the sample an absorbance scan (300–800 nm; 1 nm steps) of the supernatant was made using a VARIAN Cary 50 photo spectrometer.

### Plant growth and acetylene reduction assay

In this study *A. evenia* (CIAT22838; Malawi), *A. deamii* (LSTM #24; Mexico), *A. tambacoudensis* (LSTM #60; Senegal), and *A. sensitiva* (LSTM #28; Senegal) were used as model NF-independent *Aeschynomene* species and *A. afraspera* (LSTM #1; Senegal), and *A. nilotica* (IRRI 014040; Senegal) as model NF-dependent ones. All seeds from above indicated *Aeschynomene* species were produced in greenhouses of our laboratory at the Laboratoire des Symbioses Tropicales et Méditerranéennes (LSTM), Montpellier, France. Sterilization of seeds, germination, growth of plants and inoculation with *Bradyrhizobium* ORS285 and derivatives were as described^24^. After 21 days post-inoculation, photos of plants were taken, the amount of nodules on the roots were counted and the acetylene reduction assay (ARA) was used to measure the nitrogenase enzyme activity^17^. In experiments to determine if supplementation of amino acids could restore the nodule organogenesis minus phenotype of the ORS285 *gltD::Tn5* mutant, amino acids (final concentration 0.5 mM) were added to the plant growth medium 1 h prior to bacterial inoculation. All plant experimental research was carried out in accordance with relevant institutional, national and international guidelines and legislation.

### Microscopy

To determine the glucuronidase activity in nodules formed by the P_GOGAT_-reporter strain, fresh nodules were embedded in 5% agarose and sectioned (50–70 µM) using a vibratome (VT1000S; Leica, Nanterre). Nodule slices were incubated for 30 min in GUS assay buffer^2^ at 37 °C. After staining the slices were washed three times with water, mounted on microscope slides and observed under bright-field illumination with a macroscope (Nikon AZ100; Champigny-sur-Marne, France).

## Supplementary Information

Supplementary Information.
