# Supplementary material for: A glutamate synthase mutant of Bradyrhizobium sp. strain ORS285 is unable to induce nodules on Nod factor-independent Aeschynomene species
Source: Sci Rep. 2021 Oct 22;11:20910. doi: 10.1038/s41598-021-00480-7 (PMC8536739; doi:10.1038/s41598-021-00480-7)
Supplement: Supplementary file 1 — Supplementary Information. [file 41598_2021_480_MOESM1_ESM.pdf]

Supplementary information

A glutamate synthase mutant of *Bradyrhizobium* sp. strain ORS285 is unable to induce nodules on Nod factor-independent *Aeschynomene* species

Nico Nouwen[1], Clémence Chaintreuil[1], Joel Fardoux[1] and Eric Giraud[1]

[1] Laboratoire des Symbioses Tropicales et Méditerranéennes (LSTM), UMR IRD/SupAgro/INRAE/Université de Montpellier /CIRAD - Campus de Baillarguet, Montpellier, France

Corresponding author: nico.nouwen@ird.fr

Table S1. Growth of *Bradyrhizobium* ORS285 and derivatives on BNM-B minimal medium plates containing 10 mM succinate as carbon source and the indicated compound (10 mM) as nitrogen source. Growth was analysed after 7 days at 28°C. - : no growth ; + : pin colonies ; ++ : normal growth ; +++ : very good growth.

**Nitrogen-source**	**ORS285**	**ORS285 *gltD::Tn5***	**ORS285 *gltD::Tn5* + *gltD***	**ORS285 *gltD::Tn5* + *E. coli gdhA***
KNO_3	+++	-	+++	-
NH_4Cl	+++	-	+++	+++
urea	+++	-	+++	+++
Alanine	+++	-	+++	++
Arginine	+++	-	+++	+
Asparagine	+++	++	+++	++
Aspartic acid	+++	++	+++	++
Cysteine	-	-	-	-
Glutamic acid	+++	++	+++	++
Glutamine	+++	++	+++	+++
Histidine	+++	+	+++	++
Isoleucine	++	++	++	++
Methionine	-	-	-	-
Leucine	+++	+++	+++	+++
Proline	+	-	+	+
Serine	+++	-	+++	++
Threonine	-	-	-	-
Tryptophan	+++	-	+++	++
Valine	-	-	-	-

Table S2. Strains used in this study.

**Strain**	**Description**	**Source**
Bradyrhizobium		
ORS285	WT strain	[1]
ORS285 *gltD::Tn5*	*gltD* gene inactivated by insertion of Tn5 transposon	This work
ORS285 *gltD::Tn5* + *gltD*	*gltD::Tn5* gene of ORS285 *gltD::Tn5* repaired by insertion pJG194-*gltB-gltD*	This work
ORS285 *gltD::Tn5* + *gdhA*	ORS285 *gltD::Tn5* strain with insertion of pJG194-$P_{BRADO4694}$-*gdhA*	This work
ORS285 P_{GOGAT}-*gusA*	Reporter strain to measure the activity of the *gltB-gltD* promoter region	This work
Escherichia coli		
XL2 Blue	Strain used for cloning	Agilent
S17-1	Donor strain for conjugation	[2]
MG1655	Strain used for cloning the *E. coli gdhA* gene	[3]

Table S3. Plasmids used in this study.

Plasmid	Description	source
pGEM-T Easy	Plasmid used for cloning PCR fragments	Promega
pJG194	Plasmid used for inactivating / introducing genes in rhizobia	[4]
pJG194-4694-miaA	pJG194 with transcriptional fusion promoter region BRADO4694 and *miaA* gene	[5]
pJG194-*nodA-gusA*	pJG194 with transcriptional fusion promoter region *nodA* gene ORS285 and *gusA*	[6]
pJG194-*gltB-gltD*	pJG194 with 3' region of *gltB* and complete *gltD* gene of ORS285	This work
pJG194-*gdhA*	pJG194 with transcriptional fusion promoter region BRADO4694 and *gdhA* gene of *E. coli* MG1655	This work
pJG194-P_{GOGAT}-*gusA*	pJG194 with transcriptional fusion promoter region *gltB-D* operon and *gusA*	This work

Table S4. Oligonucleotide primers used in this study.

**Primer couples**	**Sequence (5’ → 3’)**	**usage**
gltB_F	ACTAGTGGTCGACGTGTTCAAGAACC	PCR amplification 3’ region *gltB* gene and complete *gltD* gene and cloning as *Spe*I-*Bam*HI fragment
gltD_R	GGTGGTGGATCGGATCCAACTAAG	
gdhA_F	GGATCCTACGGCGAACAATGCTCGACTCAC	PCR amplification *E. coli* MG1655 *gdhA* gene and cloning as *Bam*HI-*Eco*RI fragment
gdhA_R	GAATTCTTAAATCACACCCTGCGCCAGCATCG	
P_{GOGAT}_F	GTCGACCGAGACGGCTCCAACTAGCAACTTC	PCR amplification promoter region *gltB-D* operon and cloning as *Sal*I-*Bam*HI fragment
P_{GOGAT}_R	GGATCCATGAAGCCGACGCCGCAGGAATCCT	

References

1. Molouba, F. *et al.* Photosynthetic bradyrhizobia from *Aeschynomene* spp. are specific to stem-nodulated species and form a separate 16S ribosomal DNA restriction fragment length polymorphism group. *Appl. Environ. Microbiol.* **65**, 3084–3094 (1999).

2. Simon, R., Priefer, U. & Pühler, A. A broad host range mobilization system for *in vivo* genetic engineering: transposon mutagenesis in Gram negative bacteria. *Bio/Technology* **1**, 784–791 (1983).

3. Blattner, F. R. *et al.* The complete genome sequence of Escherichia coli K-12. *Science* **277**, 1453–1462 (1997).

4. Griffitts, J. S. & Long, S. R. A symbiotic mutant of *Sinorhizobium meliloti* reveals a novel genetic pathway involving succinoglycan biosynthetic functions. *Mol. Microbiol.* **67**, 1292–1306 (2008).

5. Podlešáková, K. *et al.* Rhizobial synthesized cytokinins contribute to but are not essential for the symbiotic interaction between photosynthetic bradyrhizobia and *Aeschynomene* legumes. *Mol. Plant-Microbe Interact.* **26**, 1232–1238 (2013).

6. Nouwen, N., Arrighi, J. F., Gully, D. & Giraud, E. RibBX of *Bradyrhizobium* ORS285 plays an important role in intracellular persistence in various *Aeschynomene* host plants. *Mol. Plant-Microbe Interact.* **34**, 88–99 (2021).

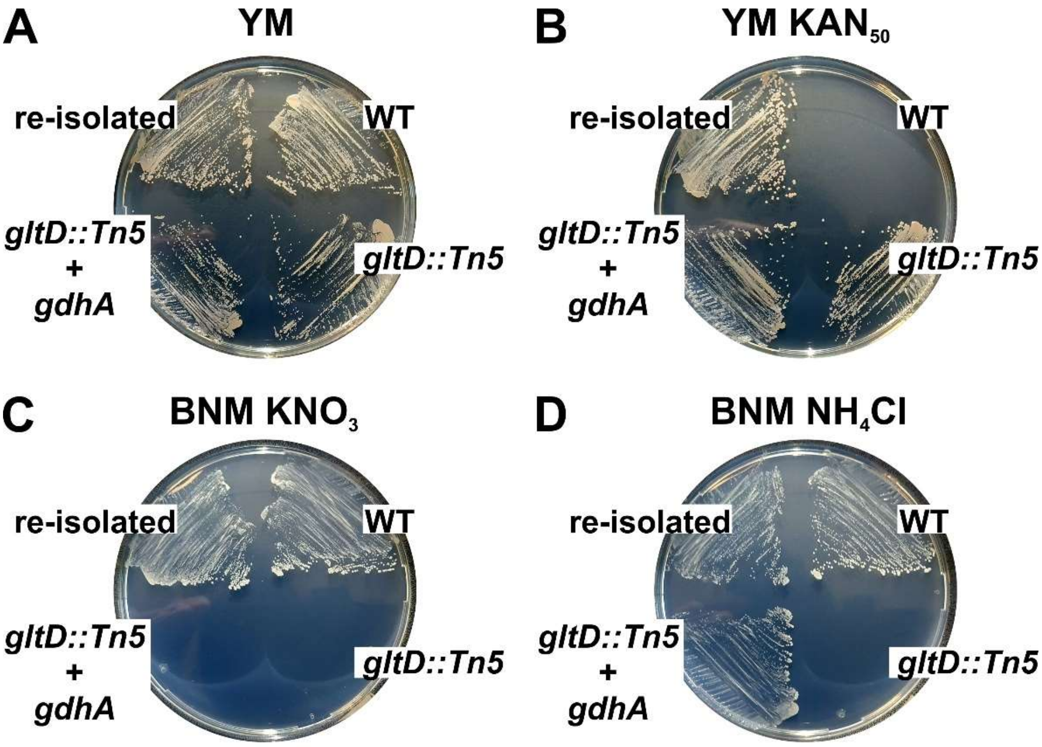

Figure S1

Fig. S1. *Bradyrhizobium* ORS285 *gltD::Tn5* + *E. coli gdhA* mutant cells re-isolated from nodules of *A. evenia* plants have obtained the capacity to grow on Minimal Medium plates containing potassium nitrate as nitrogen source. Wild-type ORS285, ORS285 *gltD::Tn5*, ORS285 *gltD::Tn5* + *E. coli gdhA* and a representative ORS285 *gltD::Tn5* + *E. coli gdhA* bacterium re-isolated from *A. evenia* nodules grown on (**A**) YM plates, (**B**) YM plates plus 50 µg/ml kanamycin, (**C**) BNM-B Minimal medium plates containing 10 mM ammonium chloride and (**D**) BNM-B Minimal medium plates containing 10 mM potassium nitrate as nitrogen source. Growth on plates was analysed after 7 days at 34°C.

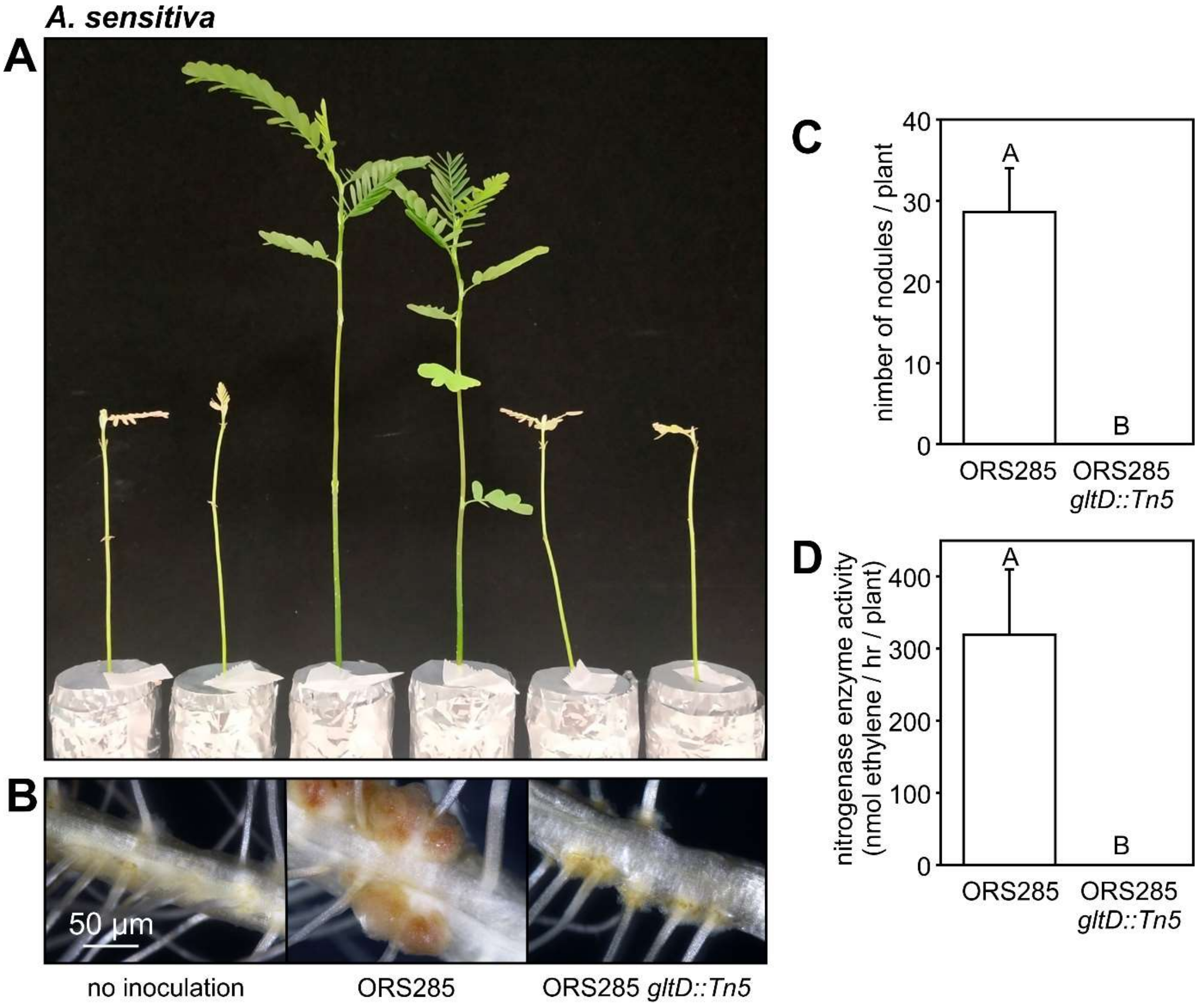

Figure S2

Fig. S2. The *Bradyrhizobium* ORS285 *gltD::Tn5* mutant does not induce nodules on *Aeschynomene sensitiva* (LSTM #28) plants. (**A**) Comparison of the growth of *A. sensitiva* plants inoculated with ORS285 and ORS285 *gltD::Tn5*. Non-inoculated plants (ni) were used as control. (**B**) Roots of *A. sensitiva* plants inoculated with ORS285 and ORS285 *gltD::Tn5.* (**C**) Number of root nodules on *A. sensitiva* plants inoculated with ORS285 and ORS285 *gltD::Tn5,* respectively. The mean number of nodules per plant (n = 5) at 21 dpi is presented. (**D**) Acetylene reducing activity of *A. sensitiva* plants inoculated with ORS285 and ORS285 *gltD::Tn5* at 21 dpi. The mean amount of produced ethylene per hour and per plant (n = 5) is indicated. In (**C**) and (**D**) error bars represent standard errors of the mean and letters represent conditions with significant difference according to the Tukey's test ($P < 0.05$).

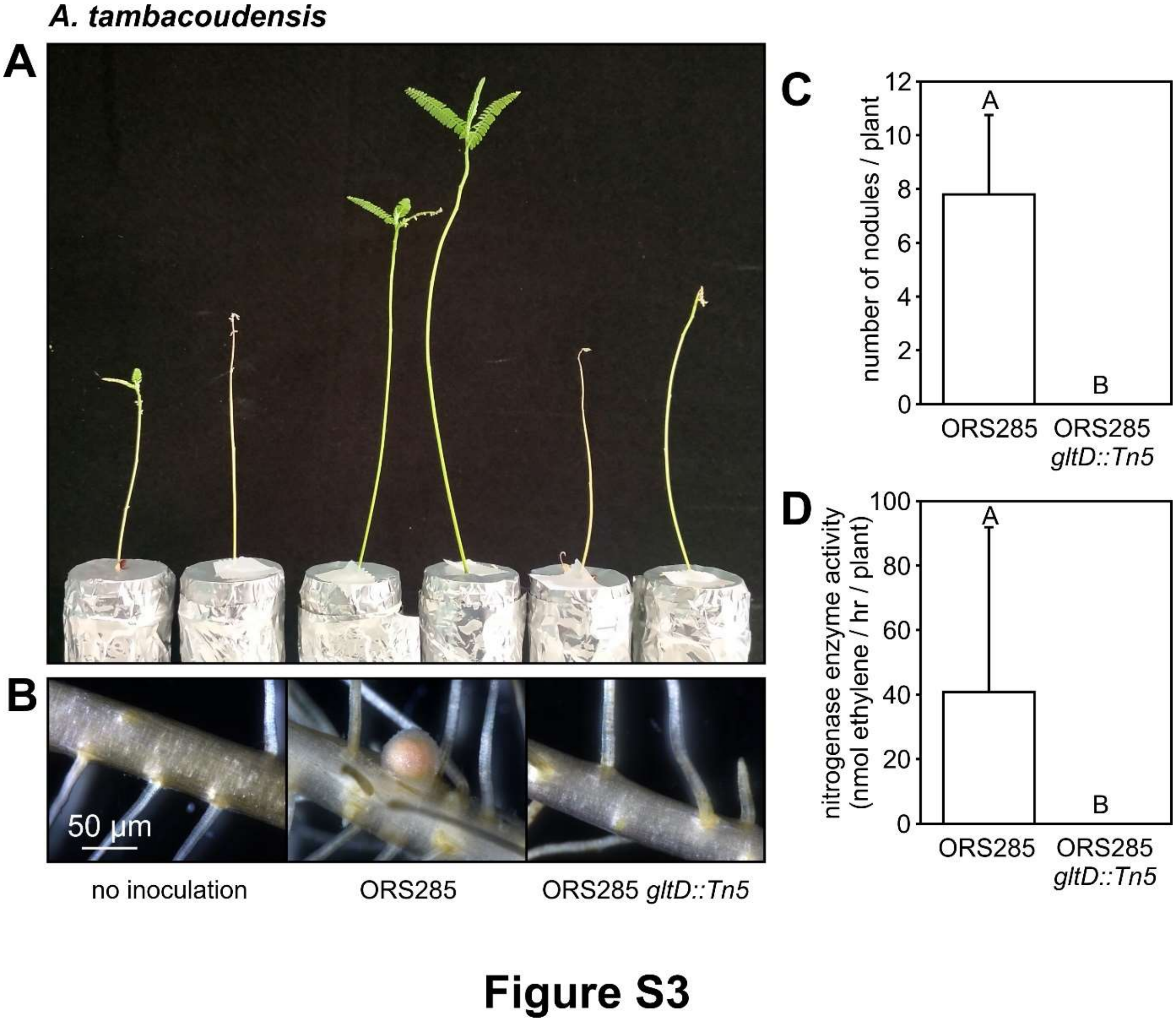

Fig. S3. The *Bradyrhizobium* ORS285 *gltD::Tn5* mutant does not induce nodules on *Aeschynomene tambacoudensis* (LSTM #60) plants. (**A**) Comparison of the growth of *A. sensitiva* plants inoculated with ORS285 and ORS285 *gltD::Tn5*. Non-inoculated plants (ni) were used as control. (**B**) Roots of *A. tambacoudensis* plants inoculated with ORS285 and ORS285 *gltD::Tn5.* (**C**) Number of root nodules on *A. tambacoudensis* plants inoculated with ORS285 and ORS285 *gltD::Tn5,* respectively. The mean number of nodules per plant (n = 5) at 21 dpi is presented. (**D**) Acetylene reducing activity of *A. tambacoudensis* plants inoculated with ORS285 and ORS285 *gltD::Tn5* at 21 dpi. The mean amount of produced ethylene per hour and per plant (n = 5) is indicated. In (**C**) and (**D**) error bars represent standard errors of the mean and letters represent conditions with significant difference according to the Tukey's test ($P < 0.05$).

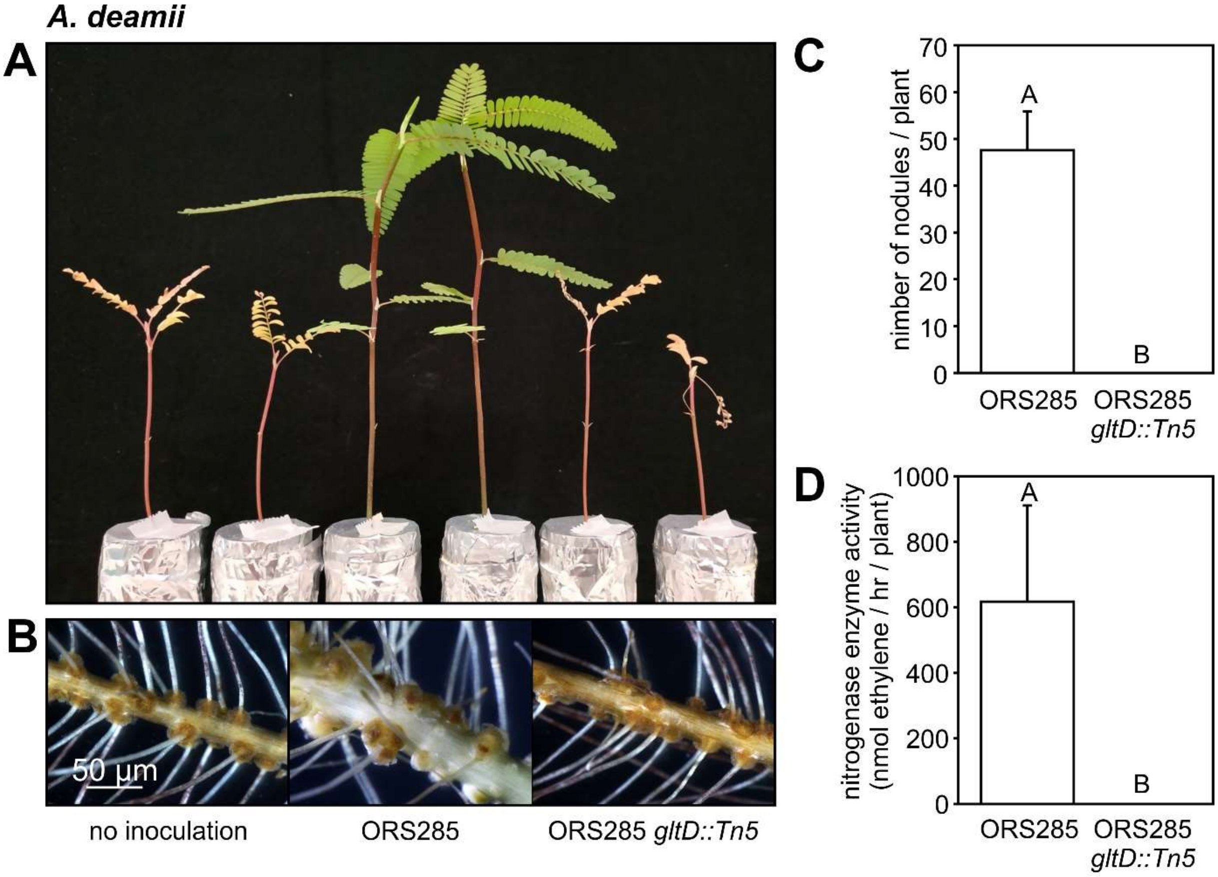

Figure S4

Fig. S4. The *Bradyrhizobium* ORS285 *gltD::Tn5* mutant does not induce nodules on *Aeschynomene deamii* (LSTM #24) plants. (**A**) Comparison of the growth of *A. deamii* plants inoculated with ORS285 and ORS285 *gltD::Tn5*. Non-inoculated plants (ni) were used as control. (**B**) Roots of *A. deamii* plants inoculated with ORS285 and ORS285 *gltD::Tn5*. (**C**) Number of root nodules on *A. deamii* plants inoculated with ORS285 and ORS285 *gltD::Tn5,* respectively. The mean number of nodules per plant (n = 5) at 21 dpi is presented. (**D**) Acetylene reducing activity of *A. deamii* plants inoculated with ORS285 and ORS285 *gltD::Tn5* at 21 dpi. The mean amount of produced ethylene per hour and per plant (n = 5) is indicated. In (**C**) and (**D**) error bars represent standard errors of the mean and letters represent conditions with significant difference according to the Tukey's test ($P < 0.05$).

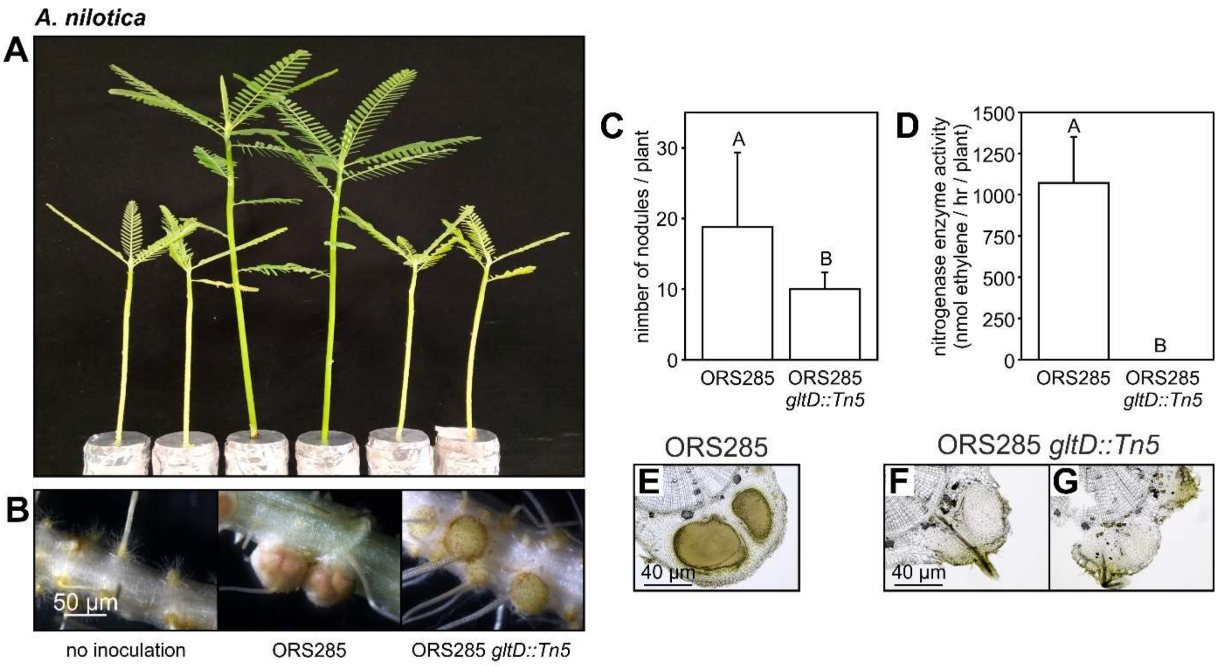

Figure S5

Fig. S5. The *Bradyrhizobium* ORS285 *gltD::Tn5* mutant does induce nodules on *Aeschynomene nilotica* (IRRI 014040) plants. (**A**) Comparison of the growth of *A. nilotica* plants inoculated with ORS285 and ORS285 *gltD::Tn5.* Non-inoculated plants (ni) were used as control. (**B**) Roots of *A. nilotica* plants inoculated with ORS285 and ORS285 *gltD::Tn5*. (**C**) Number of root nodules on *A. nilotica* plants inoculated with ORS285 and ORS285 *gltD::Tn5*, respectively. The mean number of nodules per plant (n = 5) at 21 dpi is presented. (**D**) Acetylene reducing activity of *A. nilotica* plants inoculated with ORS285 and ORS285 *gltD::Tn5* at 21 dpi. The mean amount of produced ethylene per hour and per plant (n = 5) is indicated. In (**C**) and (**D**) error bars represent standard errors of the mean and letters represent conditions with significant difference according to the Tukey's test (P < 0.05). 70 µM nodule sections of *A. nilotica* plants inoculated with (**E**) wild-type ORS285 and (**F**), (**G**) ORS285 *gltD::Tn5* mutant. Note the presence of a gold-brown substance in the nodule tissue and inside some plant cells in nodule sections of *A. nilotica* plants inoculated with the ORS285 *gltD::Tn5* mutant strain.
